# Threats Posed by the Fungal Kingdom to Humans, Wildlife, and Agriculture

**DOI:** 10.1128/mBio.00449-20

**Published:** 2020-05-05

**Authors:** Matthew C. Fisher, Sarah J. Gurr, Christina A. Cuomo, David S. Blehert, Hailing Jin, Eva H. Stukenbrock, Jason E. Stajich, Regine Kahmann, Charles Boone, David W. Denning, Neil A. R. Gow, Bruce S. Klein, James W. Kronstad, Donald C. Sheppard, John W. Taylor, Gerard D. Wright, Joseph Heitman, Arturo Casadevall, Leah E. Cowen

**Affiliations:** a MRC Centre for Global Infectious Disease Analysis, Imperial College, London, United Kingdom; b Department of Biosciences, University of Exeter, Exeter, United Kingdom; c Infectious Disease and Microbiome Program, Broad Institute of MIT and Harvard, Cambridge, Massachusetts, USA; d U.S. Geological Survey, National Wildlife Health Center, Madison, Wisconsin, USA; e Department of Microbiology and Plant Pathology, Center for Plant Cell Biology, Institute for Integrative Genome Biology, University of California—Riverside, Riverside, California, USA; f Max Planck Fellow Group Environmental Genomics, Max Planck Institute for Evolutionary Biology, Plön, Germany; g Environmental Genomics, Christian-Albrechts University, Kiel, Germany; h Max Planck Institute for Terrestrial Microbiology, Department of Organismic Interactions, Marburg, Germany; i Department of Molecular Genetics, University of Toronto, Toronto, Ontario, Canada; j The National Aspergillosis Centre, Wythenshawe Hospital, The University of Manchester, Manchester Academic Health Science Centre, Manchester, United Kingdom; k Department of Biosciences, University of Exeter, Exeter, United Kingdom; l Department of Pediatrics, Department of Internal Medicine, and Department of Medical Microbiology and Immunology, School of Medicine and Public Health, University of Wisconsin—Madison, Madison, Wisconsin, USA; m Michael Smith Laboratories, University of British Columbia, Vancouver, British Columbia, Canada; n McGill Interdisciplinary Initiative in Infection and Immunology, Departments of Medicine, Microbiology & Immunology, McGill University, Montreal, Canada; o University of California—Berkeley, Department of Plant and Microbial Biology, Berkeley, California, USA; p M.G. DeGroote Institute for Infectious Disease Research, Department of Biochemistry and Biomedical Sciences, DeGroote School of Medicine, McMaster University, Hamilton, Ontario, Canada; q Department of Molecular Genetics and Microbiology, Medicine, and Pharmacology and Cancer Biology, Duke University Medical Center, Durham, North Carolina, USA; r Department of Molecular Microbiology and Immunology, Johns Hopkins Bloomberg School of Public Health, Baltimore, Maryland, USA; s The Donnelly Centre, University of Toronto, Toronto, Ontario, Canada; t RIKEN Center for Sustainable Resource Science, Wako, Saitama, Japan; Vallabhbhai Patel Chest Institute

**Keywords:** antifungal resistance, biodiversity, food security, fungal pathogens, global health, medical mycology, plant-pathogenic fungi, wildlife pathogens

## Abstract

The fungal kingdom includes at least 6 million eukaryotic species and is remarkable with respect to its profound impact on global health, biodiversity, ecology, agriculture, manufacturing, and biomedical research. Approximately 625 fungal species have been reported to infect vertebrates, 200 of which can be human associated, either as commensals and members of our microbiome or as pathogens that cause infectious diseases. These organisms pose a growing threat to human health with the global increase in the incidence of invasive fungal infections, prevalence of fungal allergy, and the evolution of fungal pathogens resistant to some or all current classes of antifungals. More broadly, there has been an unprecedented and worldwide emergence of fungal pathogens affecting animal and plant biodiversity. Approximately 8,000 species of fungi and Oomycetes are associated with plant disease. Indeed, across agriculture, such fungal diseases of plants include new devastating epidemics of trees and jeopardize food security worldwide by causing epidemics in staple and commodity crops that feed billions. Further, ingestion of mycotoxins contributes to ill health and causes cancer. Coordinated international research efforts, enhanced technology translation, and greater policy outreach by scientists are needed to more fully understand the biology and drivers that underlie the emergence of fungal diseases and to mitigate against their impacts. Here, we focus on poignant examples of emerging fungal threats in each of three areas: human health, wildlife biodiversity, and food security.

## Abstract (precis)

The fungal kingdom includes at least 6 million eukaryotic species and is remarkable with respect to its profound impact on global health, biodiversity, ecology, agriculture, manufacturing, and biomedical research. Approximately 625 fungal species have been reported to infect vertebrates, 200 of which can be human associated, either as commensals and members of our microbiome or as pathogens that cause infectious diseases. These organisms pose a growing threat to human health with the global increase in the incidence of invasive fungal infections, prevalence of fungal allergy, and the evolution of fungal pathogens resistant to some or all current classes of antifungals.

## INTRODUCTION

The fungal kingdom includes millions of species (1), ranging from the largest organism on earth (2) to a myriad of microscopic species, some of which are pathogenic for plants and animals. For many fungi, the capacity to sense environmental cues and transition between distinct morphologies has a profound impact on their ability to reproduce, forage for nutrients, invade tissues, and evade immune responses (3, 4). In the biosphere, fungi occupy a critical role as degraders of organic matter, which allows the release and reuse of elements and nutrients from dead organisms (5). This ability to degrade organic matter, and in particular plant remains, requires complex enzymatic suites that are also relevant for their role as pathogens because many of those same proteases, phospholipases, cellulases, hemicellulases, pectinases, ligninase, and nucleases can play important roles in virulence. Fungi are also remarkable as factories of secondary metabolites that include such important medical compounds as antibiotics and immunosuppressants, as well as mycotoxins that can be deleterious to human, animal, and plant health. Although fungi were historically thought to be related to plants based on their production of cell walls and the morphology of mushrooms, molecular analyses in the 1990s showed that they are closer to animals (6). This phylogenetic relationship hinders the discovery of antifungal drugs to treat human and animal diseases because it implies that there are fewer metabolic differences suitable for targets in drug development.

In nature, fungi exist in symbiotic relationships with such diverse species as plants and algae, to form mycorrhizae and lichens, respectively. Fungi often reside in complex communities composed of multiple cell types, with biofilms as a predominant life form (7). Fungi are important members of diverse microbiota and promote ecosystem homeostasis through interactions with bacteria in lungs, guts, soil, stems, and other environments (8–10). Although fungi are generally considered auxotrophic, they can also harvest electromagnetic radiation for growth, which implies some autotrophic capacity (11–13). Fungi are highly resilient and capable of successfully occupying extreme environments. For example, the damaged radioactive reactor at Chernobyl hosts dozens of melanotic fungal species, despite high radiation fluxes (14), and the Antarctic fungus Cryomyces antarcticus has been shown to survive under space station simulated Mars conditions (15). Fungi hold the record for growth at elevated temperature among eukaryotic organisms; Aspergillus fumigatus, the major human lung allergen and pathogen, is able to grow at temperatures up to 70°C and remains viable down to −20°C (16, 17). As illustrated by these examples, fungal species manifest astounding diversity, resilience, and variability with regard to the environments they occupy and their associations with other organisms.

Fungi are remarkable in their ability to adapt rapidly in response to selection pressures. Their capacity for both sexual and asexual reproduction and epigenetic change and tolerance of changes in genome ploidy underlie their ability to evolve rapidly in response to selective pressures imposed by heterogeneous and changing environments. In clinical and agricultural settings, these properties translate to an ability to readily develop resistance to antifungal agents (18). Similarly, changes in virulence and thermal tolerance can be elicited in experimental settings over short time scales. For example, coincubation of amoeba and fungal cells results in rapid increases in virulence (19–22), and progressive exposure of Metarhizium anisopliae to higher temperatures results in increased thermal tolerance that allows growth at mammalian temperatures (23). Hence, there is concern that climate change with global warming will select for new animal- and human-pathogenic fungi as species with pathogenic potential adapt to higher temperatures and thus breach mammalian thermal defenses conferred by endothermy (24). In this context, the simultaneous appearance of phylogenetically distant strains of Candida auris on three continents may be the first example of a new fungal pathogen emerging due to global warming (25).

In summary, the fungal kingdom presents tremendous opportunities and challenges to humanity through its remarkable diversity, exceptional metabolic capacity, and rapid ability to change. In the context of opportunities, the overwhelming majority of fungal species remain uncharacterized, and these could be the source of new drugs and useful compounds for industry or have synergistic roles in supporting plant growth in mineral-limited soils or resisting pathogen invasion (26). In the context of challenges, the fungal kingdom also includes numerous species with pathogenic potential that pose threats to varied ecosystems. The fact that the fungal kingdom is understudied compounds the threats posed by what has been referred to as the “hidden kingdom.” The realization that much more attention and effort need to be devoted to studying all aspects of the biology of fungi has led *Nature Microbiology* to advocate ending this neglect of fungi (27), the Royal Society to convene a meeting to strategize tackling emerging fungal threats (28), and the United States Centers for Disease Control and Prevention (CDC) to organize a fungal disease awareness week. In recognition of the importance of fungi for the biosphere, the American Academy of Microbiology held a colloquium in 2017 titled “One Health: Fungal Pathogens of Humans, Animals, and Plants” (1), which inspired this follow-up article.

## EMERGING FUNGAL THREATS TO HUMANS

The impact of fungi on human health has been underappreciated, despite the fact that these eukaryotic pathogens infect billions of people worldwide, killing in excess of 1.5 million per year (29, 30). Systemic fungal diseases were rare in humans until the 1950s, when antibiotics and the development of intensive care units revolutionized modern medicine. The 1950s also marked the start of the use of immunosuppressive agents such as corticosteroids, the development of cancer chemotherapies, and the inception of catheters that provide access for microbes on the body’s surface to the body’s interior. These factors enabled fungi to exploit humans in new ways. There are diverse types of fungal infections that are determined by the fungal species and the immune status of the person infected (31). Superficial infections are the most common and include the 1 billion people with skin, hair, and nail infections. Mucosal infections with *Candida* (so-called “yeast infection”) are also very common, with over 135 million women afflicted repetitively, as one example (32). More devastating are chronic, localized infections below the skin that are more common in tropical regions, as with mycetoma and chromoblastomycosis, which the World Health Organization (WHO) has recently classified as Neglected Tropical Diseases. Likewise, chronic fungal lung infections, notably with *Aspergillus* spp., are complications of tuberculosis and other chronic pulmonary diseases, affecting millions globally (33). Fungal allergy is also common, with millions affected, worsening asthma and cystic fibrosis or leading to chronic nasal and sinus symptoms (33). Invasive fungal infections are progressive and lethal if not diagnosed and specifically treated. These infections are initiated by inhalation or inoculation of spores or by mobilization of commensal fungi that reside within the body. Although a few species of fungi have the capacity to cause disease even in healthy people, most invasive fungal infections are caused by fungi with low pathogenic potential that cause disease only in patients with defects in innate or acquired immunity, including in the context of premature births, HIV infection, and severe influenza, as well as due to immunosuppressive treatments for cancer transplantation. These invasive fungal diseases impose a major public health burden, with mortality rates of 30% to 90%, depending on the pathogen and patient population.

Approximately 90% of deaths due to fungal infection are caused by species of *Aspergillus*, *Cryptococcus*, *Candida*, or *Pneumocystis*, with additional threats posed by fungi such as *Coccidioides* and *Histoplasma*, which can infect even immunocompetent hosts in regions of endemicity (29, 30). An acute threat is posed by *Aspergillus* species, with more than 30 million people at risk of invasive aspergillosis each year because of corticosteroid or other treatments and over 300,000 patients developing this infection annually (34). A. fumigatus is a common culprit, associated with an overall 50% mortality rate even if diagnosed and treated swiftly. There is a growing appreciation that *Aspergillus* and other fungi can be a major cause of life-threatening allergy associated with severe asthma (35, 36). *Cryptococcus* species are also now recognized as one of the most significant fungal threats to human health (37). The WHO places the annual global burden of cryptococcal meningitis in excess of 223,100 cases annually, causing more than 181,000 deaths in patients living with HIV and approximately 15% of all AIDS-associated deaths. Cryptococcosis is caused almost exclusively by two closely related species that are commonly encountered in the environment: Cryptococcus neoformans and Cryptococcus gattii (38–40). Another AIDS-defining illness has been *Pneumocystis* pneumonia, which remains a common infection in resource-limited regions and has expanded to diverse patient populations, including transplant recipients and individuals afflicted with lung disease, autoimmune disease, or cancers of the blood or lymph systems (41). In the context of hospital-acquired invasive fungal infections, species of *Candida* are the most prevalent causal agent, with Candida albicans as the most frequent culprit and associated with mortality rates approaching 40% despite state-of-the-art treatment options (42). Current blood culture methods, the usual means of diagnosing systemic infection, are only 40% sensitive. More sensitive molecular approaches, such as β-glucan detection or PCR testing, are costly and not widely available, leading to an underestimation of the burden of invasive candidiasis. The CDC has classified *Candida* as a serious threat to human health due to the dramatic rise in drug-resistant infections, especially those caused by non-*albicans* species (43). Overall, there is a growing concern regarding the impact of outbreaks of fungal diseases.

An emerging fungal pathogen that has gained international attention is Candida auris, which is typically multidrug resistant and frequently implicated in life-threatening hospital-acquired infections. C. auris poses a new threat to global health due to an escalating number of cases, a high rate of antifungal resistance, and an ability to survive in hospital environments, leading to nosocomial outbreaks. Initially reported in cases of ear infections (44), this species was soon after detected as the cause of contemporaneous outbreaks in dispersed regions of the world (45, 46). Genomic analyses of these early cases identified four geographically stratified genetic subdivisions or clades (47). More recently reported outbreaks are predominantly linked to three of these clades (48–50), and a fifth potential clade was identified in 2018 from a single isolate from Iran (51). While in many countries C. auris is still a rare cause of infection, the pathogen is now responsible for a major proportion of candidemia in other regions (52).

The identification of distinct clades of C. auris in different regions of the world within the span of a few years is a striking aspect of the emergence of this species. C. auris was first identified only in 2009, and retrospective inventories of older isolate collections did not detect the species prior to 1996 (47). Owing to the high frequency of resistance to azole antifungals, exposure to these chemicals, which are used widely in both medicine and agriculture, may have played a role in the selection of azole-resistant isolates of C. auris. The ability of this yeast to colonize skin and to grow at higher temperatures is also likely to have contributed to the emergence and global dissemination of C. auris. Factors that could explain how this species only recently became a global human pathogen include an increase of health care settings that may promote transmission, human interactions with the environment increasing contact with or amplifying C. auris, or rising global temperatures selecting for higher-temperature growth tolerance (25, 53). Currently, unanswered questions include where this species occurred prior to 1996, what was the prepandemic population structure, and whether there is an environmental reservoir of this fungus that spilled over to infect humans. The closest-related species of C. auris have been isolated from a variety of marine and terrestrial environments, in addition to human sources, and wide environmental surveys may help better delineate the preferred environment and provide clues as to where to search for C. auris (25, 53). It is pertinent to note that these questions broadly parallel those that were asked in the context of other fungal outbreaks, such as the global emergence of Batrachochytrium dendrobatidis in amphibians (see below), questions that culminated in the discovery of the Asian origins of amphibian chytridiomycosis 20 years after its detection (54).

While the ability of C. auris to survive in a hospital environment clearly has played a role in local outbreaks, the pathogenesis of this fungus is less clear, with contrasting reports of the virulence of this organism relative to other *Candida* species (55, 56). Infected patient populations, while not generally immunocompromised, often have other major health complications or have undergone recent invasive procedures (47). Thus, the attributable mortality related to C. auris infection is not yet clear.

Effective health care guidelines to deal with C. auris infections require better surveillance methods, rapid and accurate diagnostics, decolonization protocols, and new antifungals to combat multidrug-resistant strains. Identifying patients at high risk, in addition to those with direct contact to index cases, could be prioritized for skin colonization screening. Efforts focused on antimicrobial resistance warrant expansion to include analysis of C. auris. Wider screening would be supported by more rapid and accessible diagnostic assays, which need to differentiate C. auris from the related species Candida haemulonii. The high frequency of C. auris resistance to antifungal drugs, including isolates with reported resistance to all three major classes of antifungals used to treat invasive infections (47, 57), highlights the urgent need for the development of new antifungal therapies.

Beyond C. auris, there are major concerns for other fungal outbreaks and the rise in antifungal drug resistance. One pressing threat is posed by the ongoing outbreaks of *Trichophyton* infections in Southeast Asia that are resistant to the ergosterol biosynthesis inhibitor terbinafine (58). Strong selection for the emergence of resistance in this dermatophyte is likely attributable to the widespread use of over-the-counter medications, incomplete treatment courses, and the use of creams that combine steroids, antifungals, and antibacterials and are associated with low exposures and incomplete coverage. Another cause for serious concern has been the alarming increase in azole-resistant *Aspergillus* (18, 59–61). Selection for resistance in *Aspergillus* can occur in patients on long-term therapy, as well as in regions of intensive agricultural fungicide use, with *Aspergillus* being one of the most common globally ubiquitous eukaryotes and azoles being a dominant fungicide deployed in agriculture. The potential for azole-resistant *Aspergillus* to evolve in the field and be transmitted to the clinic has been highlighted as a major cause for concern. We have little understanding of the wider impact of the global usage of antifungal chemicals on fungi that reside in the environment.

## CONTROL STRATEGIES

A major limitation to combating the high morbidity and mortality of fungal infections is the limited application of accurate diagnostic tools. Detection of fungal infections by microscopy and culture is insensitive and often negative early in infection when treatment is most effective. There have been many recent commercial developments in nonculture diagnostics allowing immediate or within-24-h diagnosis for the most lethal infections, including cryptococcal, *Aspergillus* and *Histoplasma* antigen, *Aspergillus* antibody, and PCR tests for *Candida*, *Pneumocystis*, *Aspergillus*, Mucormycetes, and dermatophyte infections (62). Real-life experience of these tests, either singly or collectively, has demonstrated the individual and public health merits of such testing in many different clinical settings (63–67). Integration of these tests into routine care pathways in high-risk patient groups requires a major shift in thinking and allocation of health care resources (68).

Owing to the close evolutionary relationship between fungi and animals, there are a very limited number of targets for antibiotic therapy that both kill or arrest the fungus and spare the host. One antifungal target is the cell wall, which is found in fungi and is absent in animals, and the other is the cell membrane, which contains ergosterol in fungi and cholesterol in animals. Despite the many challenges in the development of safe and effective antifungals, there is recent progress in antifungal drug development (69). New members of established antifungal classes have been advanced, as evidenced by the approval of the azole inhibitor of ergosterol synthesis isavuconazole in 2015, and the ongoing clinical trials of rezafungin—a new member of the cell wall-targeting echinocandin class that does not require daily dosing. To overcome resistance and offer opportunities for better overall outcomes, drugs with novel targets are crucial. Much-needed advances in this space include olorofim, which blocks pyrimidine biosynthesis; ibrexafungerp, which inhibits β-glucan synthesis through a mechanism that is distinct from that of the echinocandins (70); and fosmanogepix, which inhibits synthesis of glycosylphosphatidylinositol-anchored cell wall mannoproteins. The potential antifungal drug target space can be further expanded by combining conventional antifungals with molecules that target key regulators of crucial responses to drug-induced cellular stress, as with the protein phosphatase calcineurin and molecular chaperone Hsp90 (71, 72). Innovative approaches that move beyond small-molecule antifungals, such as antifungal vaccines, immunotherapies, and novel antifungal biologics, are also areas of active investigation (73–76). By harnessing advances in antifungal immunity, chemical genomic screening platforms, large libraries of diverse synthetic molecules and natural products, and partnerships between academia and industry, there are exciting opportunities to develop resistance-evasive strategies to treat life-threatening and chronic fungal infections (77).

## EMERGING FUNGAL THREATS TO WILDLIFE

**FIG 1:**
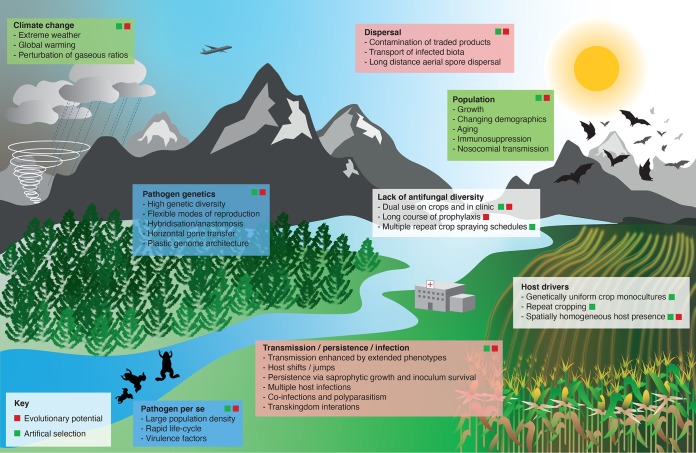
The drivers of emerging fungal threats to plants and animals. Four key factors favor the emergence of fungi that infect plants and animals. (i) Evolutionary potential (blue boxes). High pathogen abundance and virulence enhance evolutionary potential. A rapid and repeating life-cycle generates a large pathogen population density. High genetic strain diversity hastens emergence of novel fungal genotypes and lineages, with diversity generated by enhanced reproductive capacity (sexual, parasexual, or clonal), whole-genome or hyphal fusions (144), or genome plasticity endowed by aneuploidy/copy number variation (145), horizontal gene transfer (146), or two-speed genomes (147). (ii) Artificial selection and host susceptibility (gray boxes). A homogeneous host, such as a large population of animals or a crop monoculture, fuels pathogen variant emergence. Fungicide-resistant genotypes emerge due to protracted and repeated treatments/sprays and via the dual use of antifungals on crops and in the clinic (18). (iii) Dispersal/epidemiology (red boxes). Pathogens and infected hosts are disseminated globally via movement of infected biota (85), contamination of traded goods, or long-distance spore dispersal by, for example, airplanes or vectoring by hurricane winds (119, 148). Fungi can persist long-term as resting spores or via saprophytic growth. They can hop hosts or persist as generalist pathogens by causing infections on multiple hosts (18). Transmission can be enhanced by extended phenotypes (149), while coinfections, polyparasitism, and transkingdom interactions can mitigate or fuel the emergence of novel strains) (85). (iv) Environmental permissiveness (green boxes). Anthropogenic and meteorological amplifiers of fungal pathogen emergence include population growth, changing population demography fueled by urbanization or conflict, aging, immunosuppression, and nosocomial hospital-acquired infections. A warming world, with increased incidences of dramatic/extreme weather events and a change in the amounts of, for example, CO_2_ and N_2_O (150), has the potential to further fuel the emergence of fungal pathogens.

The animal kingdom is widely infected by fungi, and it is unlikely that an animal exists that is not colonized or infected by fungi. While insects are extensively infected by fungi, which are important regulators of insect populations (78), only around 625 fungi have been reported to cause infection in vertebrates (79). As with humans, resistance of vertebrate wildlife to fungi is a function of both innate and acquired immunity, bolstered by high body temperatures in endothermic mammals and birds. These factors ensure a high degree of resilience of vertebrates to the fungi that they are exposed to, and as a consequence, fungal disease in animals is often associated with compromised immunity. Nonetheless, trends have shown that emerging fungal diseases in animals are occurring worldwide (80). The factors that underlie this expansion of disease are multifactorial and are underpinned by the complex interactions among biotic and abiotic drivers described in Fig. 1. Host life-history plays an important role in determining susceptibility, and emerging fungal pathogens of wildlife such as frogs, salamanders, bats, and snakes circumvent resistance to infection mediated by high body temperature through infection of nonthermoregulating amphibians and reptiles or hibernating mammalian hosts.

Two fungal genera have acquired recent notoriety through causing very aggressive emerging infections associated with die-offs, population declines, and, in some cases, vertebrate species extinctions. These are the aquatic chytrid fungi Batrachochytrium dendrobatidis and Batrachochytrium salamandrivorans causing chytridiomycosis in susceptible species of amphibians, and the ascomycete fungus Pseudogymnoascus destructans causing white-nose syndrome (WNS) in hibernating bats of North America. In both cases, the unifying factor explaining the original emergence of these pathogens is the international dispersal of infectious inocula, which has ignited outbreaks of disease in naive host populations, followed by invasion across landscapes and host communities.

Within a year of the 1998 report of chytridiomycosis in pristine environments in Central America and Australia (81), the fungal agent was named, *Batrachochytrium dendrobatidis* sp. nov. (82), and in the ensuing 2 decades this fungus has been found infecting species across all continents except for the amphibian-free Antarctica (83). While *Batrachochytrium* was initially classified as a monotypic genus, the extirpation of fire salamanders in the Netherlands by chytridiomycosis led to the discovery of a pathogen from the same genus in 2013—*Batrachochytrium salamandrivorans* sp. nov.—proving that this genus harbored further undescribed pathogen diversity posing a further threat to amphibian biodiversity (84). Together, this class of pathogens is known as the batrachochytrids (85). To assess the impacts of batrachochytrids on global amphibian populations, Scheele et al. (86) synthesized data from multiple sources of information spanning peer-reviewed studies, the International Union for Conservation of Nature (IUCN) Red List of Threatened Species, and consultations with the scientists investigating the declines as they occurred (87) and retrospectively (88, 89). This global meta-analysis revealed that chytridiomycosis has contributed to the decline of an estimated 6.5% of extant amphibian species, including the extinction of 90 different species and representing the greatest documented loss of biodiversity attributable to any invading microorganism.

While the origins of *B. dendrobatidis* were long shrouded in mystery, recent phylogenomic analysis developed multiple lines of evidence identifying the Korean peninsula as the most likely center of origin for the panzootic (54). At least three lineages of *B. dendrobatidis* appear to have escaped out of Asia, with molecular dating showing that the expansion of the *B. dendrobatidis* Global Panzootic Lineage (*Bd*GPL) had its origins during the first half of the 20th century. The most recent out-of-Asia emergence explains the outbreak of *B. salamandrivorans* in the Netherlands following an introduction event from its endemic range in Vietnam (90). Other intercontinental and transnational introductions are occurring today for both batrachochytrids because of the global amphibian trade (54) and translocated species (91).

The outbreak of white-nose syndrome (WNS) in North American bats appears to be similarly explained by the recent introduction of this pathogen from a European center of endemism (92). The pathogen concerned, *P. destructans*, has killed millions of North American hibernating bats since emergence in New York State in approximately 2006 (93), and as of 2020, the pathogen had spread to 38 U.S. states and seven Canadian provinces (U.S. Fish and Wildlife Service; https://www.whitenosesyndrome.org/where-is-wns). The causative fungus of WNS is an obligate psychrophile that grows optimally between 12.5 and 15.8°C (54.5 and 60.4°F, respectively) with an upper growth limit of about 20°C (68°F) (94). As metabolically active bats have a body temperature of approximately 37°C, the fungal pathogen evades this limitation by infecting susceptible bat hosts during hibernation, when their core body temperatures are reduced to approximately 7°C. While mortality of hibernating bats from WNS can exceed 90% at some underground hibernation sites, for infected bats that survive until spring, emergence from hibernation can clear the fungal infection after they return to a euthermic body temperature above that conducive to growth of *P. destructans* (95).

A striking similarity between the batrachochytrids and *P. destructans* is that they are all cutaneous pathogens. While dermatophytic skin infections are typically considered to be superficial, the skin of amphibians and wing skin of bats are known to mediate complex physiological functions beyond service as a mechanical barrier, including gas exchange, electrolyte balance, and water homeostasis (96). Consequently, damage to the skin of infected amphibian and bat hosts has far-reaching physiological consequences. In amphibians, fungal skin infection has been experimentally demonstrated to cause electrolyte imbalance leading to death by cardiac arrest (97). In hibernating bats, fungal skin infection causes chronic respiratory acidosis and hyperkalemia, disrupting hibernation and inducing increased energy use in animals reliant on a strategy of energy conservation to survive winter food shortages until spring emergence (98). Thus, fungal skin infections of wildlife are not only causing catastrophic population declines but doing so through mechanisms not previously known in human or veterinary medicine, presenting novel challenges for mitigating these impacts.

## CONTROL STRATEGIES

While a sophisticated infrastructure exists to rapidly tackle outbreaks of human and agricultural pathogens, the knowledge and toolkits to combat emerging diseases in wild populations are nearly nonexistent. For instance, from the detection of batrachochytrid-driven amphibian declines through to the development of management plans and description of these disease sources took almost 3 decades (99). For WNS in bats, this timeline has been compressed, but positive outcomes continue to be hindered by limited available management options. Nonetheless, lessons have been learned and toolboxes of interventions for emerging wildlife diseases have been developed that broadly mirror the frameworks used for invasive species (100).

Management options are classified into four categories based on the stages of the pathogen invasion process—prearrival, invasion front, epizootic, and established—and are implemented as appropriate. Considering the batrachochytrids, control prearrival is focused on preventing introduction of the pathogens. Biosecurity is a first line of defense, and management actions are through strengthening import controls on disease vectors and establishment of an infection-free trade (101). For example, and motivated by the discovery of *B. salamandrivorans*, the European Union has implemented health protection measures for the trade of salamanders (102); similar measures have been adopted by the United States (103) and Canada (104). These preemergence prearrival biosecurity-oriented strategies remain the best option for avoiding disease emergence and need to be more widely adopted in the context of broadly controlling fungal pathogens of wildlife, such as bat WNS.

Following emergence along the fungal invasion front, management actions are focused on pathogen eradication, minimizing the risk of population extinction, and reducing further geographic spread. The range of management actions that are suitable for a particular pathogen-host combination can be explored through decision processes by, for instance, the use of integral projection models. This approach has been used to explore the potential control of *B. salamandrivorans*’s spread in Europe through the following management actions: (i) no action, (ii) improving host body condition, (iii) host probiotic treatments, (iv) host antifungals, and (v) host pre- and postdetection culling. Due to the high virulence of *B. salamandrivorans* in fire salamanders, the decision analysis concluded that these actions were unlikely to be effective under anything except perfect situations (105). That said, successful eradication of *B. dendrobatidis* from geographically isolated sites in Mallorca, Spain, using the antifungal itraconazole alongside an environmental disinfectant shows that sustained control can lead to the local eradication of a batrachochytrid (106). In the case of bats and WNS, strategies to control *P. destructans*, such as probiotic treatments (107), UV-mediated killing of the fungal pathogen (108), or disinfection of environmental reservoirs, have also been tested or proposed. To date, efforts to mitigate WNS have been impeded by logistical challenges of working with hibernating animals sensitive to disturbance in complex underground environments, and beneficial outcomes have not yet been clearly demonstrated.

Once established in the epizootic and established phases, management actions necessarily shift from limiting spread to reducing impacts on host populations. Options here include vaccination (109, 110) or bioaugmentation with probiotics (107, 111), manipulating habitats to favor the host over the pathogen, facilitated evolution or host-translocation of disease-resistant individuals, and *ex situ* conservation of highly threatened species. The safeguarding of threatened species through establishing *ex situ* captive breeding programs currently remains the only active conservation method to avoid species loss after invasion, and Amphibian Arks maintains the possibility for selective breeding or genetic modification of amphibians for resistance (112). Advances in gene editing represent a novel and potentially transformative tool for management of disease in free-ranging wildlife, providing possible avenues to augment host immune responses to, or attenuate virulence of, fungal pathogens (101, 113). However, as response-based control of disease in free-ranging wildlife presents challenges on numerous fronts, as with additional emerging threats such as that posed by snake fungal disease (114), investment in prevention-based strategies that support resilient wildlife populations with the capacity to adapt to health threats in the absence of routine human intervention would constitute a key component of prearrival disease control.

## EMERGING THREATS TO PLANTS

Plant-infecting fungi and oomycetes challenge the integrity of natural ecosystems and jeopardize global food security (80, 115). Indeed, we face a future blighted by known adversaries, new variants of old foes, and new diseases. Modern agricultural intensification practices, in particular, have heightened the challenge, as the planting of vast swathes of genetically uniform crops, guarded by one or two inbred resistance (R) genes, and use of single-target antifungals have accelerated the emergence of new virulent and fungicide-resistant strains. Climate change compounds the saga as we see altered disease demographics—pathogens are moving poleward in a warming world (116). It now becomes critical that we better understand how plant pathogens move in time and space and the mechanisms by which they evolve. Population data, collected over various spatial scales, are pivotal to the success of this endeavor.

### Wild species.

Fungi and oomycetes threaten the longevity and viability of natural ecosystems through infection and then decimation of wild hosts. For example, infection destroys standing trees, affecting carbon sequestration and causing habitat loss for the resident or local natural flora and fauna. While many pathogens cause persistent disease problems, such as root rot of trees, others have already changed the landscape, such as Dutch elm disease (Ophiostoma novo-ulmi) and chestnut blight (Cryphonectria parasitica) (80), and thus are considered to be largely “historical” diseases. More recently, however, there have been new disease incursions, such as myrtle rust (Austropuccinia psidii) and ash dieback (Hymenoscyphus fraxineus) (117). Both diseases are spreading around the world, challenging not only their hosts *per se* but with myrtle rust having the potential to spread to other Myrtaceae species, for example, eucalyptus and tea trees (118), and with dieback threatening not only the ash genus but also other family members in the Oleaceae (117).

### Crops.

Global food insecurity, caused by infection and subsequent decimation of our crops, comes in two guises, as it derives from (i) pre- or postharvest loss of calorie crops—that is, loss of staples that provide calories for local and national consumption—and (ii) loss of commodity or cash crops, which are traded globally and where economies of whole countries depend upon export revenues to access food grown elsewhere in the world (119).

### (i) Calorie crops.

The top five global calorie crops are wheat, rice, maize, oil palm, and soybean, as derived from measuring crop yield with the metric of calories *per capita* per day (FAOSTAT 2016; http://faostat.fao.org/default.aspx). Of these, wheat and soybean harvests are currently under threat from newly emerged fungal and oomycete pathogens. They are, respectively, the wheat blast fungus, Magnaporthe oryzae; the soybean rust fungus, Phakopsora pachyrhizi (120, 121);and the soybean oomycete pathogen Phytophthora sojae (121). We focus further on the wheat blast fungus because it serves as a cautionary example regarding global trade without robust plant health legislation and highlights the urgent need for immediate data dissemination via “open science,” by the international team of scientists involved (122).

The wheat blast fungus Magnaporthe oryzae (also known as Pyricularia graminis-tritici) was first described in 1985 in Brazil. It subsequently spread across central and southern Brazil (1988) and has since been reported in Bolivia, Paraguay, and Argentina (1996 to 2007) (123). In 2016, the fungus arrived in southwestern Bangladesh (124) and has since devastated the wheat harvest in eight districts, with total loss of crop and accounting for the loss of circa 3.5% of the total Bangladeshi wheat harvest (abstracted from 2016 USDA Foreign Agricultural drive BG6005; https://www.fas.usda.gov/data/Bangladesh-grain-and-feed-annual). The sudden arrival of wheat blast posed a series of questions. Where had it come from? Would the epidemic spread both within the country and to neighboring countries? Would disease impact the daily Bangladeshi diet by loss of calories? Could disease-resistant cultivars be deployed for planting? A consortium of United Kingdom, Bangladeshi, Swiss, and French scientists joined forces in a field pathogenomics and population genetics study to gather, sequence, and analyze the pathogen on diseased field-harvested wheat leaves (122). Their combined phylogenomics expertise led to the conclusion that the fungus had been introduced into Bangladesh from South America (124). Their findings were validated when “rotten wheat” was reported to have been imported from Brazil into Bangladesh in 2015 (124), attesting to poor phytosanitary practices at both point of export and point of import. Such international engagement, with sequence data released quickly onto an open-science web-based platform (http://www.Wheatblast.net), was unprecedented in the field of plant-pathogen studies.

This disease incursion and its outcome highlight four key points: (i) the need for avid disease surveillance, that is, scrutiny of imported goods and in the field, particularly under environmental conditions conducive to disease spread; (ii) the need for immediate data-sharing with other scientists, politicians, and the public alike for use in policy making; (iii) the need to breed wheat resistant to wheat blast infection, whether by traditional breeding (e.g., by global breeding programs as coordinated by CIMMYT, the International Maize and Wheat Improvement Center), by identification of R genes, or via CRISPR-edited wheat lines (122); and (iv) the need to understand the changing profile of local diets, such as the increasing amount of wheat eaten in Bangladesh (now ∼18% of the daily diet), both homegrown and from rising wheat imports.

### (ii) Commodity crops.

The top commodity crops are far harder to define than calorie crops (125). However, here we consider bananas as a major cash crop, on which the livelihoods of tens of thousands of those dwelling in Africa, Central and South America, and Asia depend, and focus on the challenge of Panama disease of bananas caused by Fusarium oxysporum f. sp. *cubense* Tropical Race 4 (TR4).

Bananas (*Musa* spp.) are the most consumed, the cheapest, and the most traded global fruit (126). They are, in fact, the fifth most traded agricultural product. While ca. 100 billion bananas are thought to be consumed each year (Bananalink, https://www.bananalink.org.uk/), precise figures on global banana production are elusive, as many are grown by smallholder farmers, particularly in Africa, Asia, and Latin America and are traded and consumed locally. Commercial bananas are triploids that do not produce mature seeds and are propagated vegetatively (127). There are hundreds of different varieties worldwide (127), but the most widely traded banana, from the Cavendish subgroup, accounts for approximately 47% of global production, with ca. 100 million tons produced globally each year (FAOSTAT, www.fao.org). As such, this is a precious cash or commodity crop, particularly in countries such as Ecuador, Philippines, and Costa Rica. To illustrate this point, in terms of food security, Costa Rica exports ∼13% of the world’s trade in bananas, and the revenue generated covers ∼40% of Costa Rica’s food imports (abstracted from *http://www.fao.org/economic/est/est-commodities/bananas*).

Cavendish bananas are widely planted and dominate the U.S. and European Union import markets, being higher yielders, more resilient to extremes of weather, and more robust in transit than other cultivars. However, their hugely restricted genetic basis is a matter of grave concern for global banana production. Indeed, the longevity of the Cavendish banana is jeopardized by the spread of an aggressive fungus, F. oxysporum f. sp. *cubense* (Fusarium odoratissimum) TR4. Infection of Cavendish and many other banana varieties (126) causes wilting, leaf chlorosis, vascular discoloration, and loss of crop. TR4 was first described in Malaysia, Indonesia, and China (1990s) and then Australia (1997), Jordan and Mozambique (2013), Europe (2018), and Turkey (2019) (128), and its first incursion into Latin America, notably Colombia, was recorded in 2019 (129). This fungus persists in the soil or on decaying host plant debris, with chlamydospores allegedly remaining viable for up to 30 years (126). TR4 invades susceptible roots and causes severe vascular wilt disease. Descriptions of root infection and subsequent colonization of the rhizome (130, 131), and progression of the fungus through to the pseudostem (132) and aerial tissues (133), have generated greater understanding of its infection cycle. There remain, however, many unknown details regarding both the biology of whole-plant infection and the pathogen characteristics that drive its spread.

Current efforts involve sequencing of F. oxysporum f. sp. *cubense* populations, including TR4 and other races from different geographical locations. A goal of population genomic analyses will be to decipher the origin and spatiotemporal migration routes in time and space of the pathogen and to identify the race-specific mutations or genomic regions that define TR4. Genome sequencing of other F. oxysporum *forma specialis* fungi has revealed that this group of fungi comprise highly dynamic genomes (134, 135). For example, the genome of the tomato-infecting lineage F. oxysporum f. sp. *lycopersici* includes highly variable lineage-specific chromosomes encoding virulence determinants that are essential for virulence. F. oxysporum propagates mainly by clonal reproduction; however, lineage-specific chromosomes can be exchanged horizontally between strains (135). Thereby, new virulence determinants can be acquired or exchanged between strains by vegetative fusion. It is possible that the virulence specificities of TR4 and other F. oxysporum f. sp. *cubense* races likewise are encoded by lineage-specific chromosomes.

## CONTROL

There are currently no commercial banana cultivars resistant to TR4. Interestingly, wild banana species surveyed in natural forests of Indonesia in proximity to banana plantations infected by TR4 showed no symptoms of *Fusarium* wilt (136). This observation points to wild banana species as a potential source of *Fusarium* wilt resistance genes. Improving disease resistance in commercial banana cultivars will be an important way forward as there are no effective fungicides described to date, and thus, disease control can currently be affected only by quarantine and hygiene.

Current global disease control for crops largely relies on chemical application that threatens the health of humans and animals, negatively impacts ecosystems, and generates drug-resistant plant-pathogenic strains. Innovative and ecofriendly fungicides and antimicrobial agents are in urgent need to avoid uncontrollable outbreaks of pathogen and parasite infections in both plants and animals, including humans. There may also be opportunities for development of biological control strategies with environmental microbes that produce pathogen-specific antimicrobial agents.

One alternative rapid approach is to utilize cross-kingdom RNA interference (RNAi) to express double-stranded RNAs (dsRNAs) or small RNAs (sRNAs) that target pathogen or insect virulence-related genes in plants to combat plant diseases (137–139). This approach can also control multiple pathogens simultaneously by designing dsRNA and sRNA constructs that target genes from different pathogens (140). Furthermore, the recent discovery of fungal RNA uptake (140) makes it possible to develop spray-induced gene silencing (SIGS) to control fungal pathogens through topical application of pathogen gene-targeting dsRNAs or sRNAs (140–142). Spray application of long dsRNAs or sRNA duplexes that target fungal virulence-related genes can effectively suppress infection of Botrytis cinerea and Sclerotinia sclerotiorum (140, 142) on fruits, vegetables, and flowers and control Fusarium graminearum infection on the monocot barley (141). Such RNA-based, “new-generation” fungicides may serve as an effective alternative for disease control by targeting any pathogen that can take up RNAs and has effective RNAi machinery and for which important virulence genes are known.

Finally, changing the architecture of agricultural ecosystems by increasing diversity may carry the potential to reduce disease pressure on crops (143). Species mixtures, as opposed to monocultures, reduce the spread of fungal pathogens within fields and may thereby also slow down the evolution of new virulence traits. Further, mixed-cropping systems have the potential to increase soil microbial diversity, including mutualistic symbionts that add to improved plant health. Challenges of mixed-cropping systems will be to alleviate yield damage and to develop technical solutions to manage crop diversity.

## THE WAY FORWARD

In summary, it is clear that interdisciplinary research networks are needed to tackle four grand challenges presented by fungi: (i) understand the forces driving the emergence, evolution, and spread of fungi affecting plants, animals, humans, and society; (ii) identify mechanisms of fungal adaptation and interactions with hosts and other microbes; (iii) understand the evolution of resistance to fungicides and antifungals across the fungal kingdom; and (iv) implement current successful diagnostic approaches and develop novel strategies to thwart fungal disease. With human activity, modern medicine, and environmental and climate change all intensifying the impact of fungi (29, 80), it is now crucial to mobilize such networks to solve the most pressing threats to global health, agriculture, and biodiversity.
